# Automated alert and activation of medical emergency team using early warning score

**DOI:** 10.1186/s40560-021-00588-y

**Published:** 2021-12-07

**Authors:** Soo Jin Na, Ryoung-Eun Ko, Myeong Gyun Ko, Kyeongman Jeon

**Affiliations:** 1 Department of Critical Care Medicine, Samsung Medical Center, Sungkyunkwan University School of Medicine, Seoul, Republic of Korea; 2 Intensive Care Unit Nursing Department, Samsung Medical Center, Sungkyunkwan University School of Medicine, Seoul, Republic of Korea; 3 Division of Pulmonary and Critical Care Medicine, Department of Medicine, Samsung Medical Center, Sungkyunkwan University School of Medicine, 81 Irwon-ro, Gangnam-gu, Seoul, 06351 Republic of Korea

**Keywords:** Hospital rapid-response team, Hospital emergency service, Clinical alarm, Physiologic monitoring, Instrumentation

## Abstract

**Background:**

Timely recognition of warning signs from deteriorating patients and proper treatment are important in improving patient safety. In comparison to the traditional medical emergency team (MET) activation triggered by phone calls, automated activation of MET may minimize activation delays. However, limited data are available on the effects of automated activation systems on the time from derangement to MET activation and on clinical outcomes. The objective of this study was to determine the impact of an automated alert and activation system for MET on clinical outcomes in unselected hospitalized patients.

**Methods:**

This is an observational study using prospectively collected data from consecutive patients managed by the MET at a university-affiliated, tertiary hospital from March 2013 to December 2019. The automated alert system automatically calculates the Modified Early Warning Score (MEWS) and subsequently activates MET when the MEWS score is 7 or higher, which was implemented since August 2016. The outcome measures of interest including hospital mortality in patients with MEWS of 7 or higher were compared between pre-implementation and post-implementation groups of the automated alert and activation system in the primary analysis. The association between the implementation of the system and hospital mortality was evaluated with logistic regression analysis.

**Results:**

Of the 7678 patients who were managed by MET during the study period, 639 patients during the pre-implementation period and 957 patients during the post-implementation period were included in the primary analysis. MET calls due to abnormal physiological variables were more common during the pre-implementation period, while MET calls due to medical staff’s worries or concern about the patient’s condition were more common during the post-implementation period. The median time from deterioration to MET activation was significantly shortened in the post-implementation period compared to the pre-implementation period (34 min vs. 60 min, *P* < 0.001). In addition, unplanned ICU admission rates (41.2% vs. 71.8%, *P* < 0.001) was reduced during the post-implementation period. Hospital mortality was decreased after implementation of the automated alert system (27.2% vs. 38.5%, *P* < 0.001). The implementation of the automated alert and activation system was associated with decreased risk of death in the multivariable analysis (adjusted OR 0.73, 95% CI 0.56–0.90).

**Conclusions:**

After implementing an automated alert and activation system, the time from deterioration to MET activation was shortened and clinical outcomes were improved in hospitalized patients.

**Supplementary Information:**

The online version contains supplementary material available at 10.1186/s40560-021-00588-y.

## Introduction

Hospital ward patients often show abnormal physiological signs hours before adverse events such as unexpected cardiac arrest [1]. Timely recognition of warning signs from deteriorating patients and proper treatment are important in improving patient safety. Thus, many hospitals around the world adopted rapid response systems such as medical emergency teams (MET), which include specially trained staff members and systems to respond to deteriorating patients [2]. However, previous studies provided controversial evidence for the effects of MET [3, 4]. One reason for discrepancies in the results is weak points in the rapid-response system, such as decreased sensitivity for detecting clinical deterioration and delays in MET activation [5].

Activation of the MET is initiated by identifying patients who are deteriorating or at risk of deterioration [6]. The use of a physiological tracking and trigger systems to monitor all patients in an acute hospital is recommended [7]. An automated processing system can improve accuracy and reliability for the detection of deterioration and, therefore, is a desirable feature of monitoring systems [8]. In comparison to the traditional MET triggered by phone calls, automated activation of MET may minimize activation delays [9–12]. Accordingly, we hypothesized that the implementation of an automated alert and activation system of MET would improve the early activation of MET for clinically deteriorating patients and result in improved patient outcomes. However, limited data are available on the effects of automated activation systems on the time from derangement to MET activation and on clinical outcomes in unselected hospitalized patients in a general ward. Therefore, we investigated the characteristics of MET activation and patient outcomes before and after implementing an automated alert and activation system for MET.

## Methods

All consecutive patients who were managed by the MET were prospectively registered following the initiation of the MET program at Samsung Medical Center (a 1989-bed university-affiliated, tertiary referral hospital in Seoul, South Korea), which provides care for approximately 92,000 inpatients per year. To address the primary research question of whether implementation of an automated alert and activation system for MET is associated with clinical outcomes in hospitalized patients, we compared clinical data on patients managed by MET before and after implementing the automated alert and activation system in August 2016. The institutional review board of the Samsung Medical Center approved this study and waived the requirement for informed consent because of the observational nature of the research. Additionally, patient information was anonymized and de-identified prior to analysis.

### Study population

All consecutive adult patients who were managed by the MET on the general ward between March 1, 2013 and December 31, 2019 were included in the study. Some clinical data for patients enrolled between 2013 and 2018 were included in a previous study [13]. Since the active screening program with an automated alert and activation system was only implemented in the general ward, patients outside of the general ward, such as the outpatient department or day care unit were excluded from the study. In addition, MET calls for which outcome data were not available were excluded. If patients were repeatedly managed by the MET during the same episode of hospitalization, the first event was used as an index MET activation.

**Fig. 1:**
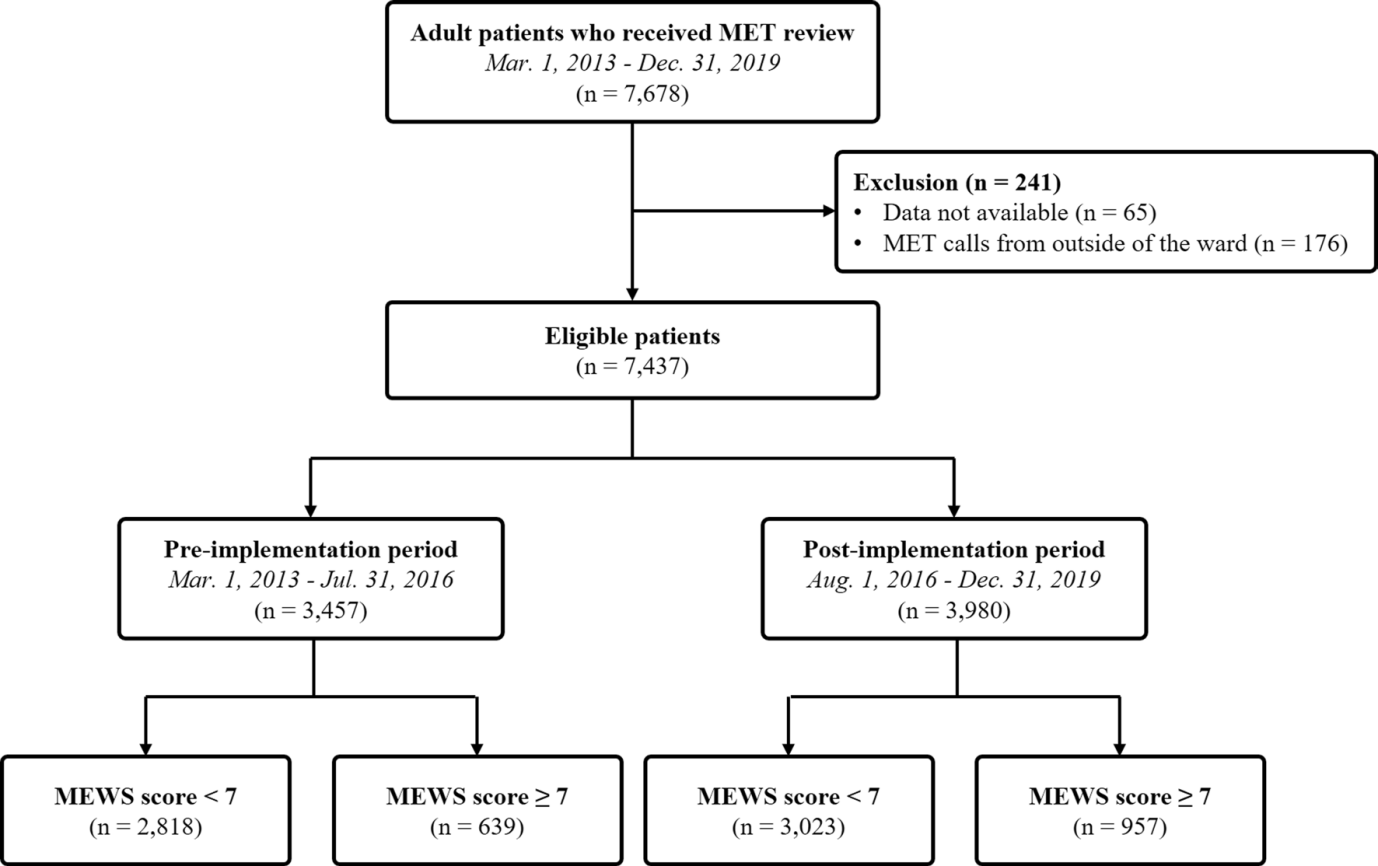
Scheme for patient enrollment. *MET* medical emergency team

To address the primary research objective of evaluating the effect of the automated alert and activation system with the Modified Early Warning Score (MEWS) [14], only patients with MEWS of 7 or higher at the time of MET activation were included in the primary analysis. The final patient cohort was divided into the pre-implementation period (before August 2016) and the post-implementation period (after August 2016) (Fig. 1).

### Operation of the MET

Details of the hospital’s MET system have been described in previous publications [13, 15, 16]. The hospital-wide MET at the Samsung Medical Center was introduced at the beginning of March 2009. Since March 2013, the MET consists of dedicated intensivist physicians, including critical care fellows and attending physicians, which provide round-the-clock coverage. Before implementation of the automated system, physicians and nurses directly contacted the MET using a dedicated phone number when a patient met any single criterion (see Additional file 1). Activation was also allowed when the medical staff was concerned about changes in their patient’s clinical condition, even in the absence of physiological disorders that meet the criteria. An automated alert and activation system was integrated into the original MET activation process in August 2016. Calls for MET activation were available for all patients regardless of do-not-resuscitate status during the study period.

When activated, the MET is expected to arrive within 10 min, complete patient assessments within 30 min, and order diagnostic tests and therapeutic treatments relevant to the patient’s condition. After assessment and therapeutic interventions by the MET, patients who are considered to require treatment and monitoring that cannot be provided outside of the ICU are transferred to the ICU, while patients in a stable condition remain on the general ward.

### Implementation of the automated alert and activation system

In August 2016, the MET initiated an active screening program for all ward patients using the automated alert and activation system based on a MEWS. The MEWS was automatically calculated using five physiological parameters (systolic blood pressure, heart rate, respiratory rate, body temperature, and level of consciousness) when nurses recorded the patient’s vital signs into the electronic medical record. If MEWS was 7 or higher, an automated alert was sent to MET members as a text message in real-time, 24 h a day, 7 days a week, and the MET was activated automatically. Information about the patient’s code status was recorded in the hospital electronic medical record and patients who consented to a do-not-resuscitate order were excluded from the active screening. Alerts were not displayed to physicians and nurses of the ward, but color-coded MEWS were displayed in the patient list in the electronic medical record interface. Thus, medical staff of the ward could recognize the patient’s MEWS status: green for MEWS 0–2, yellow for MEWS 3–4, orange for MEWS 5–6, and red for MEWS ≥ 7. Patient vital signs were recorded at the bedside immediately after measurement using a laptop or portable device whenever possible. The frequency of measuring vital signs was made according to the order of the attending physician without a prescribed hospital policy, but vital signs were usually measured at least four times a day and more frequently if the patient’s clinical condition changed. MEWS was automatically updated whenever a vital sign was newly recorded.

### Data collection and clinical outcomes

Details of all MET activations triggered by call and automated alert were recorded in the specified format as soon as possible after the event by a MET member in a registry. The following information was collected and recorded: patient demographics, method that triggered MET (call or automated alert), reasons for the MET activation based on calling criteria, time of the first documented physiological disorder, MEWS, time of the MET activation and deactivation, vital signs at the time of the MET activation and deactivation, interventions delivered by the MET, and the patient’s disposition after the clinical episode [6, 17]. These data were supplemented on the next day with a retrospective review of hospital medical records before registration for quality control of registry data.

When the MET was activated by the automated alert and activation system, the physiologic derangements of the patient at the time of the MET activation were recorded as reasons for MET activation based on calling criteria with physiologic variables with specific cut-offs (see Additional file 1). If there was no physiologic derangement that satisfies the calling criteria, it was classified as a call due to concern about overall deterioration. The time points were defined as follows: the time of the first documented physiological derangement was the first time at least one of the criteria for MET activation was met; the time of MET activation was the time when the MET was triggered by call or automated alert; the time of MET deactivation was the admission time to the ICU when the patient was transferred from the general ward or the time when the patient’s disposition was finally determined. The duration of the MET intervention was from activation to deactivation.

The primary outcome of this study was hospital mortality. Secondary outcomes included unplanned ICU admissions and hospital length of stay. Data on clinical outcomes were collected through a retrospective review of hospital medical records. Patients who decided to limit treatment after MET intervention were excluded from outcome analyses because self-imposed treatment limitations could affect the clinical outcomes [18].

### Statistical analysis

Descriptive statistics were performed to compare the characteristics and clinical outcomes between the two periods before and after implementing the automated alert and activation system. Continuous variables were expressed as medians and interquartile ranges and were compared with a Mann–Whitney *U* test. Categorical variables were summarized with numbers and percentages and were analyzed using Chi-squared tests or Fisher’s exact tests, where applicable. A logistic regression method was used to determine the odds ratios of the active screening with the automated alert and activation system and to identify the risk factors for hospital mortality in ward patients with clinical deterioration. Continuous variables were converted into binary variables for logistic regression using operating characteristic curve analysis with the Youden Index to determine the optimal cut-off point [19]. The implementation of the automated alert and activation system, and variables with a *P*-value less than 0.1 on univariate analyses [20], as well as a priori variables that were clinically relevant, were entered into the forward stepwise multiple logistic regression model [21]. The results were reported as odds ratios (ORs) of each variable with 95% confidence intervals (CIs). A two-tailed *P*-value of less than 0.05 was considered statistically significant for all analyses. Data were analyzed using STATA version 14.0 statistical software (Stata Corp, College Station, TX, USA).

## Results

During the study period, 7678 consecutive patients were managed by the MET. Among them, 176 patients outside of the general ward and 65 patients whose outcome data were not available were excluded. Finally, 7437 eligible patients were retrieved and 639 (18.5%) of 3457 patients during the pre-implementation period and 957 (24.1%) of 3980 patients during the post-implementation period with MEWS of 7 or higher were included in the primary analysis (Fig. 1).

### Characteristics of patients and MET activation

The characteristics of all patients and MET activation in the pre-implementation and post-implementation periods are shown in Table 1. The majority of patients managed by the MET were medical patients, in which the most common primary diagnosis for hospital admission was hemato-oncologic disease (48.7%). MET calls were more common on weekdays than on weekends and at nighttime rather than daytime. The percentage of MET calls on weekdays was higher in the post-implementation period than in the pre-implementation period. In both periods, the most common reason for MET calls was derangement of the circulatory system. The MET calls that met the criteria related to the respiratory (56.3% vs. 41.3%, *P* < 0.001) and neurologic systems (16.3% vs. 10.8%, *P* = 0.001) were higher in the pre-implementation period, while MET calls due to concern about overall deterioration (3.4% vs. 10.3%, *P* < 0.001) were higher in the post-implementation period. Median MEWS scores of patients reviewed by MET during the pre-implementation and post-implementation period were 8 and 7, respectively (*P* = 0.001). The median time from derangement to MET activation was significantly shorter in the post-implementation period (34 min vs. 60 min, *P* < 0.001). In the post-implementation period, application of high-flow nasal cannula or non-invasive ventilation by the MET was more common. Only advice was given in more cases in the post-implementation period.

**Table 1:** Patient clinical characteristics and MET activation

Characteristics	Pre-implementation (*n* = 639)	Post-implementation (*n* = 957)	*P*-value
Age, years	62 (52–72)	62 (51–71)	0.316
Male	374 (58.5)	570 (59.8)	0.627
Medical department	503 (78.7)	738 (77.1)	0.451
Primary diagnosis for hospital admission			
Gastrointestinal diseases	49 (7.7)	70 (7.3)	0.792
Cardiovascular diseases	14 (2.2)	15 (1.6)	0.361
Pulmonary diseases	47 (7.4)	82 (8.6)	0.384
Kidney diseases	8 (1.3)	10 (1.0)	0.701
Hemato-oncologic diseases	311 (48.7)	467 (48.8)	0.960
Infectious diseases	35 (5.5)	49 (5.1)	0.754
Other medical diseases	39 (6.1)	45 (4.7)	0.219
General surgery	50 (7.8)	110 (11.5)	0.017
Neurosurgery	14 (2.2)	25 (2.6)	0.593
Thoracic surgery	4 (0.6)	13 (1.4)	0.163
Other surgical disease	68 (10.6)	71 (7.4)	0.025
Activation day and time			
Weekday	432 (67.6)	709 (74.1)	0.005
Daytime hours (08:00–17:59)	288 (45.1)	404 (42.2)	0.259
Reason for MET call			
Respiratory system	360 (56.3)	395 (41.3)	< 0.001
Circulatory system	467 (73.1)	683 (71.4)	0.455
Neurologic system	104 (16.3)	103 (10.8)	0.001
Concern about overall deterioration	22 (3.4)	99 (10.3)	< 0.001
MEWS scores	8 (7–8)	7 (7–8)	0.001
Vital signs at the initiation of activation			
Heart rates, beats/min	132 (119–145)	133 (121–144)	0.510
Mean blood pressure, mmHg	72 (58–97)	73 (61–94)	0.598
Respiratory rates, breaths/min	28 (22–33)	26 (22–32)	0.021
Body temperature, °C	37.6 (36.5–38.7)	38.1 (36.6–38.8)	0.007
Time from derangement to MET activation, min	60 (17–202)	34 (4–129)	< 0.001
Interventions by MET			
Oxygen administration or increase	104 (16.4)	120 (12.7)	0.036
HFNC/NIV	14 (2.21)	49 (7.35)	< 0.001
Airway management	112 (17.7)	81 (8.5)	< 0.001
Cardiopulmonary resuscitation	25 (3.9)	20 (2.1)	0.032
Cardioversion	9 (1.4)	14 (1.5)	0.926
Bolus fluid administration	224 (35.3)	200 (21.1)	< 0.001
Medication therapy	243 (38.3)	397 (41.9)	0.159
Advice or consultation only	85 (13.4)	357 (37.5)	< 0.001
Treatment limitation	72 (11.3)	107 (11.2)	0.957
Duration of MET intervention, min	65 (39–116)	62 (36–132)	0.953

Values are given as the median (interquartile range) or *n* (%)

*HFNC* high-flow nasal cannula, *MET* medical emergency team, *MEWS* Modified Early Warning Score, *NIV* non-invasive ventilation

### Clinical outcomes

**Fig. 2:**
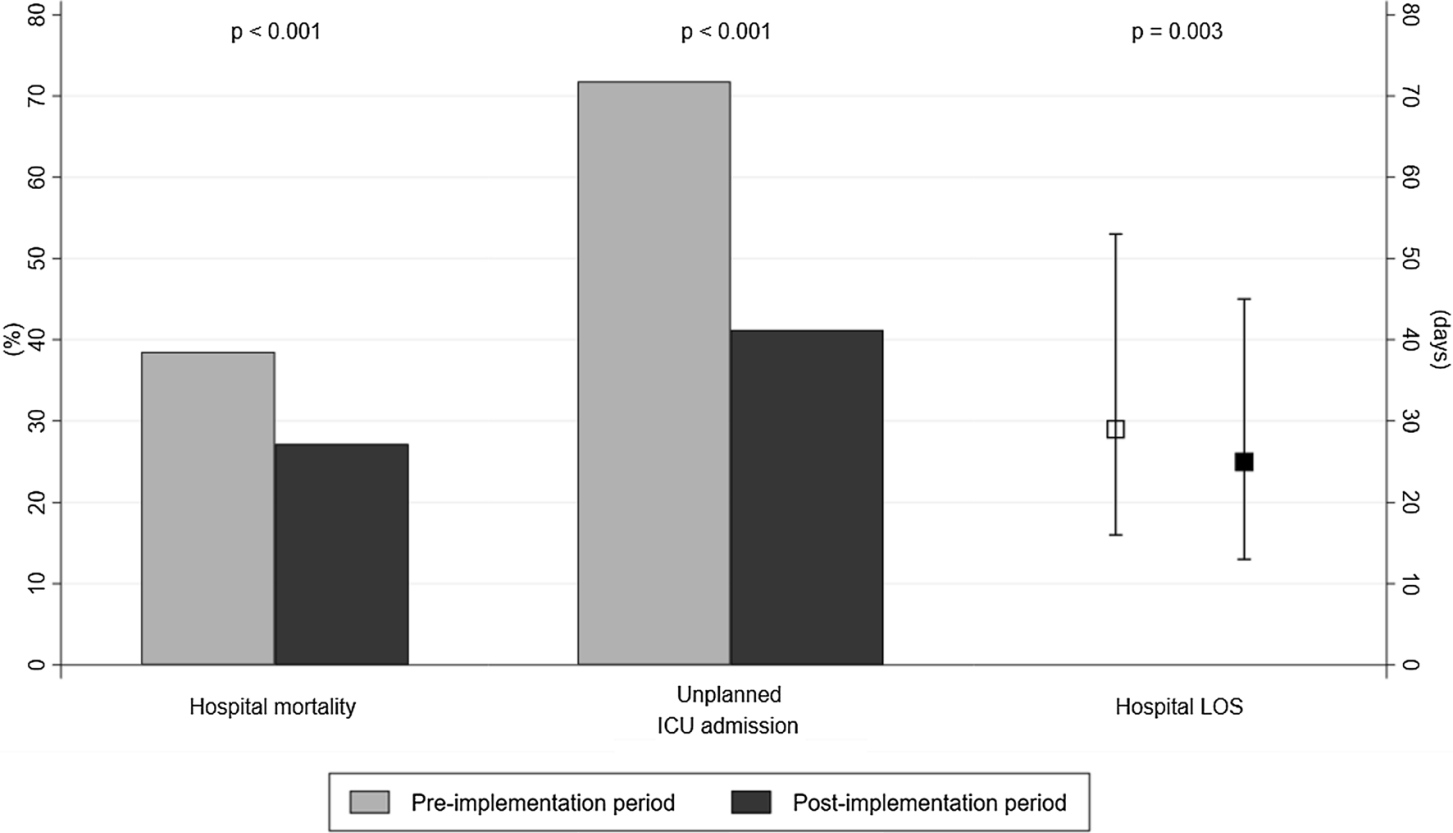
Clinical outcomes according to MEWS scores. Hospital mortality, unplanned ICU admissions, and hospital length of stays. Gray and black bars represent percentages in the pre-implementation period and post-implementation period, respectively. Gray and black squares indicate the median values for the pre-implementation period and post-implementation period, respectively. Error bars indicate the interquartile range for the 25th and 75th percentiles. *ICU* intensive care unit, *LOS* length of stay

After excluding patients who decided to limit treatment, 218 (38.5%) patients in the pre-implementation period and 231 (27.2%) patients in the post-implementation period died during hospitalization (*P* < 0.001) (Fig. 2). Compared to the pre-implementation period, unplanned ICU admission rates (41.2% vs. 71.8%, *P* < 0.001) and hospital length of stay (25 days vs. 29 days, *P* = 0.003) were reduced during the post-implementation period (Fig. 2).

### Prognostic factors for hospital mortality

The univariate logistic regression analysis indicated that 19 characteristics of patients and MET activation were associated with hospital mortality. In the multivariate analysis, medical department, admission for hemato-oncologic diseases, time from derangement to MET activation, respiratory system as a reason for MET activation, HFNC/NIV, and cardiopulmonary resuscitation were associated with increased risk of death, while implementation of the automated alert and activation system (adjusted OR 0.73, 95% CI 0.56–0.90, *P* = 0.018), fever at the time of activation (adjusted OR 0.44, 95% CI 0.34–0.57, *P* < 0.001), bolus fluid administration (adjusted OR 0.58, 95% CI 0.43–0.78, *P* < 0.001) and medication therapy during MET activation (adjusted OR 0.74, 95% CI 0.56–0.96, *P* = 0.026) were associated with decreased risk of death (Table 2).

**Table 2:** Predictors of MET calls for hospital mortality

Characteristics of MET calls	Adjusted OR	95% CI	*P*-value
Implementation of automated alert system	0.73	0.56–0.95	0.018
Age > 70	1.26	0.96–1.65	0.103
Medical department	1.49	1.03–2.16	0.036
Hemato-oncologic diseases	2.89	2.17–3.85	< 0.001
MET activation during weekend	1.19	0.92–1.54	0.194
MET activation during nighttime hours	1.00	0.78–1.26	0.968
Time from derangement to MET activation, 10 min	1.01	1.00–1.01	0.004
Reason for MET activation			
Respiratory system	1.46	1.10–1.93	0.009
Circulatory system	1.16	0.84–1.58	0.366
Bedside concern	0.65	0.32–1.30	0.220
Vital sign at the time of MET activation			
Hypotension (MBP < 65 mmHg)	1.24	0.93–1.63	0.138
Tachycardia (HR > 110 beat/min)	0.95	0.63–1.43	0.797
Tachypnea (RR > 20 breath/min)	0.95	0.64–1.43	0.816
Fever (BT > 38.3)	0.44	0.34–0.57	< 0.001
MET intervention			
HFNC/NIV	1.78	1.07–2.98	0.027
Airway management	1.26	0.87–1.84	0.224
Cardiopulmonary resuscitation	5.15	2.02–13.10	0.001
Bolus fluid administration	0.58	0.43–0.78	< 0.001
Medication therapy	0.74	0.56–0.96	0.026
Advice or consultation only	0.97	0.70–1.34	0.835

*MBP* mean blood pressure, *CI* confidence interval, *HFNC* high-flow nasal cannula, *MET* medical emergency team, *MEWS* Modified Early Warning Score, *NIV* non-invasive ventilation, *OR* odds ratio

## Discussion

To our best knowledge, this is the first detailed investigation of the effects of an automated alert and activation system for MET on time from derangement to MET activation and clinical outcomes in unselected hospitalized patients on the general ward. The major finding is that implementation of such a system led to a significant decrease in time from derangement to MET activation. Moreover, unplanned ICU admissions, hospital mortality, and length of stay in those patients managed by the MET decreased after the implementation. Finally, implementation of the automated alert and activation system was independently associated with decreased risk of death in the multivariable analysis. These results suggest that an automated alert and activation system may hasten the activation of the MET and, consequently, improve outcomes in patients with MEWS score of 7 or higher.

Automated activation of the MET using electronic medical recording-based screening criteria is associated with a lower prevalence of ICU admissions [9], but the effect of automated activation on mortality has not been observed in the study. Automated activation shortened the time from derangement to MET activation, implying earlier activation. In other study, the introduction of automated alert or notification systems using predefined physiologic criteria was also associated with a reduction in standardized hospital mortality in the general medical wards [10], but the effects of shortening the time to activation on outcomes was unclear. Another automated activation of a rapid-response team using an institutional specific prediction model implemented in general medicine units was associated with a year-to-year reduction in cardiopulmonary arrest and hospital mortality [11]. Very recently, a large multicenter cohort study found that use of an automated predictive model to identify high-risk patients for whom interventions by rapid-response teams is associated with decreased mortality [12], but the effect on time to activation was not reported. Therefore, we evaluated whether the hospital-wide automated alert and activation system based on the MEWS score could shorten the time from derangement to MET activation and if this would be associated with significant improvements in clinical outcomes. Our study demonstrates that the time from derangement to MET activation was shortened and clinical outcomes, including unplanned ICU admission, hospital mortality, and lengths of hospital stays, were improved after implementing the automated alert and activation system for MET in the patients with MEWS score of 7 or higher.

To improve early recognition of unexpected deterioration, objective scores have been proposed. Most commonly, an aggregate weighted scoring system based on changes in vital signs is used. In this study, the MEWS was automatically calculated and used for automated activation of the MET. Criteria composed of only individual abnormal physiologic variables with specific cut-offs were used as triggers for MET activation during the pre-implementation period. This single parameter system had the advantage of being simple to use and reproducible, but was limited; this system did not recognize a combination of subtle changes in multiple physiologic parameters. In comparison, aggregate weighted scoring systems, such as MEWS, can recognize overall changes in patient vital signs and monitor clinical progress [14, 22]. Furthermore, aggregate scoring systems are more accurate in discriminating the risk of adverse outcomes than single parameters [23]. In this study, color-coded MEWS were displayed in the patient list in the electronic medical record interface for the medical staff to recognize overall changes in patient vital signs. And the proportion of patients who did not meet a single physiologic criterion for MET calls (according to the non-automated system) but activated MET was higher even in subgroup of patients with MEWS of less than 7 during the post-implementation period (see Additional files 2 and 3). Therefore, the introduction of MEWS increased the detection of deteriorating patients with abnormalities in multiple physiologic variables who did not meet the criteria for a single parameter. However, manual calculation of aggregate weighted scoring systems is not simple and prone to calculation errors [24]. Therefore, automated calculation and classification of the aggregate scoring system improves the recognition of patients at risk of adverse outcomes while controlling for potential errors.

An abnormal value can trigger MET activation. However, this process still requires clinician activation of the MET and, thereby, limits the automated functionality. Our alert system automatically activated MET immediately after recognizing patients with clinical deterioration. Although patient deterioration was recognized quickly, several cultural barriers to escalation of care or MET contact caused delays in MET activation [25]. In particular, bedside nurses commonly hesitate to call the MET [26]. Our center allowed both physicians and nurses to call the MET, but in practice, almost all MET calls were made by physicians. This suggests that nurses first notified the junior physician of a patient’s clinical status when abnormal vital signs were detected. The junior physician assessed the patient and then called the MET. Because the automated alert and activation system skips this process, the time from deterioration to MET activation was significantly shortened. Shortening the time from deterioration to MET activation facilitates earlier diagnosis and treatment by experienced physicians and the beneficial effects of early intervention in acutely ill patients have been identified in various clinical settings [16, 27–30].

Although this study provides additional information on automated alert and activation systems for MET in a relatively large patient cohort, our study has certain limitations that should be acknowledged. First, the study was limited by its inherent retrospective observational nature. All MET members were trained on how to record each variable and recorded the data as soon as possible after MET deactivation, but data may have been missed and recall bias may have influenced the data accuracy. Even though we performed regression modeling to control for confounders, unmeasured confounders may have been present. Second, MEWS was not used as a trigger for MET activation during pre-implementation period. Therefore, MEWS, as part of an automated alert and activation system, made it possible to cover a larger number of deteriorating cases and contributed to the improved clinical outcomes of the post-implementation period. Third, our study was conducted in a single tertiary care center, which introduces the risk of selection bias. Thus, the generalizability to other centers with different staffing, equipment, and other hospital resources may be limited. Screening methods and criteria must be tailored to the settings of each hospital to effectively implement an automated alert and activation system for MET.

## Conclusion

Implementation of an automated alert and activation system using an aggregate weighted scoring system led to a significant decrease in time from derangement to MET activation and was associated with improved clinical outcomes in the general ward. The use of an automated alert and activation program ensures rapid activation of the MET if clinical deterioration is identified; consequently, delays that occur with a non-automated system can be avoided.

## Supplementary Information

**Additional file 1: Table S1.** Calling Criteria for the Medical Emergency Team at Samsung Medical Center, Seoul, South Korea.

**Additional file 2: Table S2.** Patient clinical characteristics and MET activation in overall patients (*N* = 7437)

**Additional file 3: Table S3.** Patient clinical characteristics and MET activation in patients with MEWS < 7.

## Data Availability

The data that support the findings of this study are available on request from the corresponding author. The data are not publicly available due to privacy or ethical restrictions.

## Abbreviations

MET: Medical emergency team
ICU: Intensive care unit
MEWS: Modified Early Warning Score
CI: Confidence interval
OR: Odds ratio
