# Supplementary material for: Automated alert and activation of medical emergency team using early warning score
Source: J Intensive Care. 2021 Dec 7;9:73. doi: 10.1186/s40560-021-00588-y (PMC8650341; doi:10.1186/s40560-021-00588-y)
Supplement: Supplementary file 1 — Additional file 1: Table S1. Calling Criteria for the Medical Emergency Team at Samsung Medical Center, Seoul, South Korea. [file 40560_2021_588_MOESM1_ESM.docx]

Additional file 1

**Automated activation of medical emergency team using early warning score**

Soo Jin Na, Ryoung-Eun Ko, Myeong Gyun Ko, Kyeongman Jeon

**Table S1. Calling Criteria for the Medical Emergency Team at Samsung Medical Center, Seoul, South Korea**

| Airway and breathing | - Acute respiratory distress: respiratory rate ≥ 30 breaths/min - Acute hypoxia: oxygen saturation derived from pulse oximetry < 90% for 5 min, despite previous oxygen administration - Acute hypercapnia and acute acidosis: arterial carbon dioxide pressure > 50 mmHg and pH < 7.3 - Upper airway obstruction: stridor or use of respiratory accessory muscle |
| --- | --- |
| Circulation | - Unexplained hypotension: systolic blood pressure < 90 mmHg - Acute chest pain - Bradycardia or tachycardia: heart rate < 50 beats/min or > 130 beats/min - Arrhythmia with symptom |
| Neurology | - Sudden mental change or unexplained agitation - Seizure |
| Other | - Bedside concern about overall deterioration |
