# Supplementary material for: Automated alert and activation of medical emergency team using early warning score
Source: J Intensive Care. 2021 Dec 7;9:73. doi: 10.1186/s40560-021-00588-y (PMC8650341; doi:10.1186/s40560-021-00588-y)
Supplement: Supplementary file 2 — Additional file 2: Table S2. Patient clinical characteristics and MET activation in overall patients (N = 7437) [file 40560_2021_588_MOESM2_ESM.docx]

Additional file 2

**Automated activation of medical emergency team using early warning score**

Soo Jin Na, Ryoung-Eun Ko, Myeong Gyun Ko, Kyeongman Jeon

**Table S2. Patient clinical characteristics and MET activation in overall patients (N = 7,437)**

| Characteristics | Pre-implementation  (n = 3,457) | Post-implementation  (n = 3,980) | *P*-value |
| --- | --- | --- | --- |
| Age, years | 64 (53–73) | 64 (55–74) | 0.002 |
| Male | 1931 (55.9) | 2384 (60.0) | <0.001 |
| Medical department | 2322 (67.2) | 2652 (66.6) | 0.625 |
| Activation day and time  Weekday  Daytime hours (08:00~17:59) | 2410 (69.7)  1593 (46.1) | 2888 (72.6)  1803 (45.3) | 0.007  0.501 |
| Reason for MET call  Respiratory system  Circulatory system  Neurologic system  Concern about overall deterioration | 1465 (42.4)  1823 (52.7)  353 (10.2)  557 (16.1) | 1578 (39.7)  2031 (51.0)  333 (8.37)  747 (18.8) | 0.017  0.143  0.006  0.003 |
| MEWS scores | 4 (3–6) | 5 (3–6) | <0.001 |
| Vital signs at the initiation of activation  Heart rates, beats/min  Mean arterial pressure, mmHg  Respiratory rates, breaths/min  Body temperature, °C | 112 (93–131)  82 (66–98)  22 (20–28)  36.6 (36.3–37.5) | 115 (96–133)  80 (66–97)  22 (18–28)  36.6 (36.4–37.7) | <0.001  0.101  0.456  <0.001 |
| Time from derangement to MET activation, min | 67 (19–223) | 45 (11–147.5) | <0.001 |
| Interventions by MET  Oxygen administration or increase  HFNC/NIV  Airway management  Cardiopulmonary resuscitation  Cardioversion  Bolus fluid administration  Medication therapy  Advice or consultation only  Treatment limitation | 475 (13.8)  185 (5.4)  278 (8.1)  36 (1.0)  18 (0.5)  864 (25.1)  1190 (34.5)  990 (28.7)  206 (6.0) | 456 (11.5)  353 (12.2)  277 (7.0)  30 (0.8)  23 (0.6)  611 (15.4)  1492 (37.6)  1502 (37.8)  298 (7.5) | 0.003  <0.001  0.077  0.187  0.740  <0.001  0.006  <0.001  0.009 |
| Duration of MET intervention, min | 72 (42–136) | 63 (36–132) | <0.001 |
| Unplanned ICU admission | 1724 (53.2) | 1494 (40.7) | <0.001 |
| Hospital mortality | 928 (28.6) | 948 (25.8) | 0.009 |
| Hospital length of stay, days | 24 (13–45) | 22 (12–41) | 0.001 |

Values are given as the median (interquartile range) or n (%).

HFNC =, high flow nasal cannula, ICU = intensive care unit, MET =, medical emergency team, MEWS =, modified early warning score, and NIV =, non-invasive ventilation.
