# Supplementary material for: Automated alert and activation of medical emergency team using early warning score
Source: J Intensive Care. 2021 Dec 7;9:73. doi: 10.1186/s40560-021-00588-y (PMC8650341; doi:10.1186/s40560-021-00588-y)
Supplement: Supplementary file 3 — Additional file 3: Table S3. Patient clinical characteristics and MET activation in patients with MEWS < 7. [file 40560_2021_588_MOESM3_ESM.docx]

Additional file 3

**Automated activation of medical emergency team using early warning score**

Soo Jin Na, Ryoung-Eun Ko, Myeong Gyun Ko, Kyeongman Jeon

**Table S3. Patient clinical characteristics and MET activation in patients with MEWS < 7**

| Characteristics | Pre-implementation  (n = 2818) | Post-implementation  (n = 3023) | *P*-value |
| --- | --- | --- | --- |
| Age, years | 64 (54–73) | 65 (56–75) | <0.001 |
| Male | 1557 (55.3) | 1814 (60.1) | <0.001 |
| Medical department | 1819 (64.6) | 1914 (63.3) | 0.326 |
| Activation day and time  Weekday  Daytime hours (08:00~17:59) | 1978 (70.2)  1305 (46.3) | 2179 (72.1)  1399 (46.3) | 0.111  0.981 |
| Reason for MET call  Respiratory system  Circulatory system  Neurologic system  Concern about overall deterioration | 1105 (39.2)  1356 (48.1)  249 (8.8)  535 (19.0) | 1183 (39.1)  1348 (44.6)  230 (7.6)  648 (21.4) | 0.954  0.007  0.087  0.020 |
| MEWS scores | 4 (3–5) | 4 (3–5) | 0.302 |
| Vital signs at the initiation of activation  Heart rates, beats/min  Mean arterial pressure, mmHg  Respiratory rates, breaths/min  Body temperature, °C | 108 (90–126)  83 (68–98)  21 (19–27)  36.6 (36.3–37.3) | 110 (90–126)  82 (68–97)  21 (18–26)  36.6 (36.4–37.2) | 0.162  0.237  0.031  0.004 |
| Time from derangement to MET activation, min | 68 (20–229) | 49 (15–152) | <0.001 |
| Interventions by MET  Oxygen administration or increase  HFNC/NIV  Airway management  Cardiopulmonary resuscitation  Cardioversion  Bolus fluid administration  Medication therapy  Advice or consultation only  Treatment limitation | 371 (13.2)  171 (6.1)  166 (5.9)  11 (0.4)  9 (0.3)  640 (22.7)  947 (33.6)  905 (32.1)  134 (4.8) | 336 (11.1)  34 (13.7)  196 (6.5)  10 (0.3)  9 (0.3)  411 (13.6)  1095 (36.2)  1145 (37.9)  191 (6.3) | 0.015  <0.001  0.351  0.703  0.880  <0.001  0.038  <0.001  0.009 |
| Duration of MET intervention, min | 74 (43–139) | 63 (36–132) | <0.001 |
| Unplanned ICU admission | 1317 (49.1) | 1145 (40.5) | <0.001 |
| Hospital mortality | 710 (26.5) | 717 (25.3) | 0.336 |
| Hospital length of stay, days | 23 (12–43) | 21 (12–40) | 0.008 |

Values are given as the median (interquartile range) or n (%).

HFNC =, high flow nasal cannula, ICU = intensive care unit, MET =, medical emergency team, MEWS =, modified early warning score, and NIV =, non-invasive ventilation.
